# Navigating Prediabetes in a Foreign Country: A Qualitative Study of Self‐Management Experiences Among Chinese‐Speaking Immigrants in Australia

**DOI:** 10.1111/jan.70214

**Published:** 2025-09-22

**Authors:** Min Zhang, Kirsten J. Coppell, Johnny Lo, Lisa Whitehead

**Affiliations:** ^1^ School of Nursing and Midwifery Edith Cowan University Perth Western Australia Australia; ^2^ JBI Centre for Evidence Informed Nursing and Midwifery Perth Western Australia Australia; ^3^ Department of Medicine University of Otago Wellington Wellington New Zealand; ^4^ Nelson Marlborough Institute of Technology Nelson New Zealand; ^5^ School of Science Edith Cowan University Perth Western Australia Australia; ^6^ University of Jordan Amman Jordan; ^7^ University of Otago Christchurch Christchurch New Zealand

**Keywords:** culturally and linguistically diverse, prediabetes, reflective thematic analysis, SDG10, SDG3, self‐management

## Abstract

**Aim:**

Chinese‐speaking immigrants in Australia have a higher risk of type 2 diabetes and face more barriers to accessing quality healthcare compared to non‐culturally and linguistically diverse populations. This study aimed to explore the self‐management experiences of Chinese‐speaking Australians with self‐reported lived experience of prediabetes following immigration.

**Design:**

Qualitative study.

**Methods:**

Semi‐structured interviews were conducted with 10 purposively selected Australian Chinese‐speaking immigrants aged over 40 years. Data collection was undertaken in Perth, Western Australia between April and August 2024. Data were analysed using reflexive thematic analysis.

**Results:**

Three themes are presented in this paper: (1) An acculturation journey: Reshaping cultural identity and social connections in immigrant lives, (2) Embodying prediabetes: Cognitive reconstruction and emotional adaptation in the transition to the patient role and (3) Decision‐behaviour dynamics: Mapping agency and adaptiveness in self‐management processes. Participants demonstrated dynamic adjustment through the processes of self‐awareness, adaptive behaviours, self‐reflection and self‐attribution of health outcomes.

**Conclusion:**

Chinese‐speaking Australians navigating prediabetes following immigration underwent a complex process of reconstruction across cognitive, cultural and psychological domains. Prediabetes self‐management was shaped by cultural values, acculturation, dietary preferences, emotional resilience, local and distant social networks and resource availability. These findings underscore the importance of empowering both individuals and communities through evidence‐based and culturally appropriate strategies.

**Implications and Impact:**

Participants experienced profound transformations in their cultural adaptation, prediabetes cognition, social support networks and emotional–psychological landscape. Future interventions must address identified barriers (e.g., cooking burden, comorbidities, stress), facilitators (e.g., leisure travel, family support), motivations (e.g., cultural heritage, health risk perception) and challenges (e.g., knowledge–behaviour gap, digital health information) that shape self‐management behaviours. A community empowerment approach, utilising evidence‐based content, flexible delivery formats and existing cultural networks, should be adopted to offer promising pathways for prediabetes health education.

**Reporting Method:**

The study adhered to the Consolidated Criteria for Reporting Qualitative Research guidelines.

**Patient or Public Contribution:**

Limited patient and public involvement was incorporated, with two community representatives providing feedback on interview questions and recruitment strategies.

**Summary**

- What this paper contributes to the wider global clinical community?
  ○ Adult Chinese immigrants with prediabetes in Australia faced unique challenges in self‐management following migration, experiencing significant transformations in health cognition, cultural adaptation and social support networks. These insights may benefit other Chinese‐speaking groups or those with cultural similarities, such as those from Singapore, Taiwan, Malaysia, Indonesia and Vietnam.
  ○ The dynamic adaptive process observed in Chinese Australians navigating prediabetes enriches the patient empowerment and informs individualised interventions for promoting health and wellbeing, aligning with UN Sustainable Development Goal (SDG) 3.
  ○ The finding emphasises the necessity of community empowerment strategies to enhance prediabetes care, which could be customised for various culturally and linguistically diverse groups to address inequalities and support SDG 10.

## 1 Introduction

Australia is one of the most culturally and linguistically diverse (CALD) countries in the world (AIHW 2024). CALD is a term used to describe individuals born in non‐English speaking countries and/or who do not speak English at home (Pham et al. 2021). Significant health disparities exist between Australians from CALD backgrounds and their non‐CALD counterparts, with those from CALD backgrounds having higher rates of diabetes risk factors, diabetes, diabetes hospitalisations and mortality; yet, they frequently face considerable challenges in navigating the healthcare system, such as lower health literacy, language barriers and a lack of culturally appropriate care (e.g., Diabetes Australia 2023). These disparities result from multiple factors spanning their home country and Australia, including not only socio‐cultural, economic, environmental and genetic factors, but also their migration experience (AIHW 2022).

Type 2 diabetes and its precursor, prediabetes, have reached epidemic levels globally (IDF 2021). By 2045, the number of people living with prediabetes is projected to increase by 36% to reach 1.1 billion, and those with diabetes by 46% (IDF 2021). In Australia, about one in six adults aged over 25 years has prediabetes. Without sustained lifestyle changes, approximately 5%~10% of people with prediabetes progress to type 2 diabetes annually (Tabák et al. 2012). By 2050, the number of people with diabetes in Australia is projected to reach 3.6 million, a 2.5‐fold increase from the current prevalence (Diabetes Australia 2023). Despite this prediction, in Australia, there is currently no systematic approach to type 2 diabetes prevention and no effective strategies for high‐risk groups from CALD backgrounds (Diabetes Australia 2023), including Chinese‐speaking immigrants.

## 2 Background

Chinese‐speaking immigrants who account for 5.5% of Australia's population (ABS 2021) have higher rates of type 2 diabetes and elevated diabetes‐related mortality rates compared to the general population (O'Callaghan et al. 2022). As the largest CALD group in Australia, Chinese‐speaking immigrants—especially the 25% with limited English proficiency—face significant barriers to healthcare access, health literacy and lifestyle management (e.g., ABS 2021). These barriers can pose significant challenges for people with prediabetes, which requires proactive self‐management to prevent progression to type 2 diabetes. Despite this, the self‐management of prediabetes among CALD populations has received limited attention.

Prediabetes self‐management involves a collaborative process between individuals and healthcare providers to establish personalised lifestyle modification goals related to dietary change, exercise, weight loss (if overweight), alcohol restriction, smoking cessation and psychological adjustment (e.g., ADA 2025). Effective programmes like Diabetes Self‐Management Education and Support (DSMES) can prevent or delay diabetes progression (ADA 2025). However, for Chinese‐speaking immigrants living with prediabetes, self‐management involves more than the management of prediabetes but also consideration of their migration experiences, such as challenges in navigating unfamiliar healthcare systems, language barriers, acculturation and the loss of social support networks (AIHW 2022). This multifaceted approach is essential because cultural influences profoundly shape individual behaviour, creating particular challenges for migrants as they transition from their home country to their new country (ABS 2001).

Cultural and acculturation factors add complexity to self‐management behaviours. While these concepts have varied definitions, ‘culture’ is understood here as a shared set of meanings and ideas held by a group of people, and ‘acculturation’ refers to the process of adopting, acquiring and adjusting to a new cultural environment (Schumann et al. 2020; Unger and Schwartz 2012). Using Unger and Schwartz's (2012) three domains of acculturation, culture‐related concepts in this context can be categorised as: cultural values (e.g., norms, heritage), cultural practices (e.g., cross‐cultural transition, convergence) and cultural identifications (e.g., identity, belonging). Indeed, evidence from chronic disease research suggests that culture influences self‐care through illness perceptions, dietary practices, spiritual beliefs and social support systems (e.g., Osokpo and Riegel 2021). For example, traditional Chinese dietary culture values food as medicine and ‘hot‐cold’ food balance, which may conflict with Western dietary recommendations (Osokpo and Riegel 2021; Zou et al. 2022). However, these cultural influences on prediabetes self‐management require further exploration.

The self‐management of prediabetes among Chinese‐speaking immigrants is under‐researched worldwide (Zhang et al. 2024). After a thorough literature review, we identified only one cross‐sectional study on diabetes within this population group (Lin et al. 2024), which highlighted the need for culturally appropriate diabetes education materials for this group. In order to develop and implement effective and culturally appropriate prediabetes interventions for the Chinese‐speaking Australian immigrant population, it is crucial to gain an in‐depth understanding of their prediabetes self‐management challenges, immigrant and acculturation experiences and specific health needs.

## 3 The Study

### 3.1 Aim

This qualitative study aimed to explore the experiences of prediabetes self‐management among Chinese‐speaking Australians.

### 3.2 Research Question

What barriers, facilitators and cultural needs do Chinese‐Australian adults with lived experience of prediabetes face regarding the self‐management of prediabetes?

## 4 Method

### 4.1 Design

This qualitative study is part of a mixed‐methods research programme that is grounded in dialectical pluralism (Johnson 2017). A qualitative descriptive design, underpinned by constructivist principles, that aimed to understand how participants construct meaning from their prediabetes self‐management experiences, was used (Shan 2023). Semi‐structured, one‐on‐one interviews were undertaken in Perth, Western Australia, between April and August 2024. The Consolidated Criteria for Reporting Qualitative Research (COREQ) checklist (Appendix S1) and a tool for evaluating thematic analysis manuscripts for publication were used to guide this reporting (Braun and Clarke 2020; Tong et al. 2007).

### 4.2 Theoretical Framework

**FIGURE 1:**
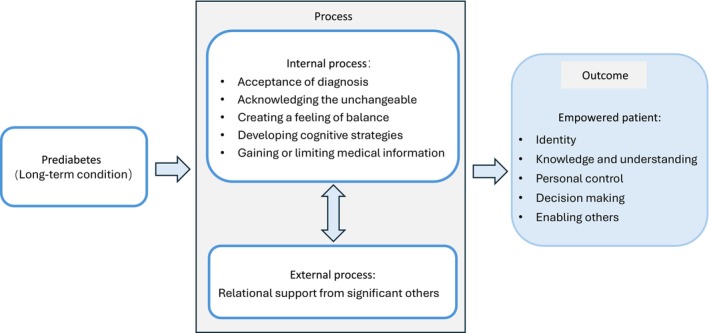
Patient Empowerment Model in prediabetes.

We adopted Small et al.'s (2013) Patient Empowerment Model (PEM) as the theoretical framework to guide the interpretation of the findings (Figure 1). This model illustrates the process and outcome of empowerment in individuals with long‐term conditions through internal and psychological cognitive processes combined with external support processes in primary care (Small et al. 2013).

### 4.3 Study Setting and Recruitment

Eleven participants were recruited from community settings in Perth, Western Australia, utilising a purposive sampling approach that combined both snowball and maximum variation techniques. This approach prioritised depth of insight and was guided by Palinkas et al.'s (2015) purposeful sampling framework which prioritises information‐rich cases over sample size while ensuring demographic diversity (Palinkas et al. 2015). Different approaches were used to recruit participants, including the distribution of bilingual flyers through Chinese community organisations, bulletin boards, social media platforms, emails, personal networks and snowball sampling. A bilingual researcher assessed eligibility and provided study information via email or WeChat. Recruitment continued until information sufficiency and rigour were reached, with no new significant insights emerging from the final interviews. Of the 11 participants recruited, one withdrew prior to the interview due to work commitments, yielding a final dataset of 10 information‐rich interview transcripts.

### 4.4 Inclusion and Exclusion Criteria

The inclusion criteria were self‐identified Chinese immigrants aged ≥ 18 years with self‐reported lived experience of prediabetes who had lived in Australia for > 6 months, spoke Mandarin or Cantonese as a first language, and were willing and able to participate. We excluded those unable to comprehend, consent to or participate in interviews.

### 4.5 Data Collection

Interview questions were informed by the research question, our scoping review findings (Zhang et al. 2024) and relevant validated instruments, including the Diabetes Self‐Management Questionnaire (Schmitt et al. 2013), the Suinn‐Lew Asian Self‐Identity Acculturation Scale (Rosenbaum et al. 2009) and the Chinese Health Beliefs scale (Eh et al. 2016). The bilingual interview schedule was professionally verified by a certified translator and reviewed by two consumer representatives, one male and one female, in collaboration with the research team (Appendix S2). After pilot testing with two participants, the question sequence was adjusted to improve clarity and flow. Participants provided written consent and selected their preferred interview arrangements, either online or at a mutually agreed‐upon location. Interviews were conducted in Mandarin by a female bilingual researcher, who employed active listening and prompts to encourage deeper insights. Interviews lasted 60–90 min and were audio‐recorded with permission, then transcribed verbatim and thoroughly checked for accuracy, including significant tones, pauses and emotional expressions. Additionally, notes were handwritten during or after each interview.

### 4.6 Data Analysis

Data analysis followed an inductive‐deductive approach using reflexive thematic analysis (RTA) (Braun and Clarke 2020). This approach was chosen for its flexibility and reflexivity, aligning with our constructivist position and research aims. The RTA approach encourages researchers to move beyond the superficial to deeply explore potential patterns and meanings within the data. We followed a six‐phase analysis process, starting with data familiarisation and followed by systematic coding (Braun and Clarke 2022). Initial line‐by‐line coding was conducted in Chinese to help preserve participants' authentic expressions and ensure data immersion. This coding approach was chosen due to the principal researcher's novice status with RTA and the richness of the data, as recommended by Saldaña (2021), Chapter 2. The second round employed more selective coding, focusing on patterns relevant to and utilised three coding techniques: in vivo coding, process coding and holistic coding.

After coding, initial themes were generated by analysing the codes for recurring patterns and shared meanings emerging from the data. These themes were then reviewed and refined through iterative analysis to accurately reflect participants' experiences. In this study, themes were developed inductively from participants' narratives, reflecting their experiences and priorities. In line with the RTA approach, we did not provide the frequencies of codes and themes. However, we included participant numbers for specific behaviours, aligning with Braun and Clarke's perspective that such counting can be ‘useful when reporting concrete practices’ (Braun and Clarke 2020). This analysis focused specifically on themes relating to the immigration experience and prediabetes self‐management in the immigrant country, aligning with our research objectives informed by our scoping review (Zhang et al. 2024). Two skilled bicultural researchers, including an external reviewer, reviewed the bilingual codes and themes to ensure interpretative rigour and cross‐cultural validity. NVivo 12.0 (Windows) was used to manage and code the data, supported by document management and visualisation tools. The PEM framework was used to guide the interpretation of the identified themes (Appendix S3).

### 4.7 Trustworthiness and Reflectivity

Credibility was established through regular debriefing sessions that were held with the research team; verification of interview questions by a certified translator; review of codes and themes by two bilingual researchers; and transparency regarding the researcher's role within the Chinese‐speaking community. The first author (female, MD, PhDs) engaged in personal reflexivity prior to data collection to acknowledge potential preconceptions, assumptions and personal experiences that might influence the research. Reflective documentation was maintained through memos written immediately after each interview and reflective journals documenting the ongoing reflections and self‐awareness. These were supplemented by reflective notes and thematic maps during data analysis.

Transferability was enhanced through detailed participant characteristics, transcript quotes from multiple participants, thematic maps and comparison to existing theoretical frameworks (Stalmeijer et al. 2024). To enhance dependability, initial coding and analysis were conducted in Chinese to preserve participants' original meanings. The primary researcher and an external bicultural translator then translated and compared the second‐cycle codes, subthemes and themes between the Chinese and English versions to ensure interpretive accuracy. No participant validation was conducted on transcripts or findings (Braun and Clarke 2020). Audio recordings, interview notes, raw data, transcripts, reflective documents and analysis products were kept as an audit trail.

### 4.8 Ethical Considerations

Ethical approval was obtained from the Edith Cowan University Human Research Ethics Committee on April 9, 2024 (Ethics Approval No. 2023‐04746‐ZHANG). All participants provided written informed consent and were informed of their right to withdraw at any time without any negative consequences. They were also encouraged to contact the Ethics Committee with any concerns or complaints regarding the research conduct.

## 5 Findings

### 5.1 Characteristics of Participants

The 10 participants were first‐generation immigrants aged between 44 and 69 years (Table 1). Seven were female and nine were born in Mainland China. The number of years lived in Australia ranged from 6 to 36 years. Nine participants were diagnosed with prediabetes after immigration and two had progressed to type 2 diabetes in the year prior to the interview. The duration since a diagnosis of prediabetes was an average of 5.7 years (range: 1–18 years). Most participants were married (*n* = 8) and most lived with their family (*n* = 9). Participants self‐reported a range of English proficiency, from minimal familiarity to professional expertise. Almost all participants had both public and private health insurance (*n* = 9).

**TABLE 1:** Participant characteristics (*N = 10*).

Characteristics	*n* (%)
Gender	
Female	7 (70)
Male	3 (30)
Age (years)	
44–64	7 (70)
≥ 65	3 (30)
Region of birth	
Mainland China	9 (90)
East China	3 (30)
South China	4 (40)
North China	2 (20)
Indonesia	1 (10)
Years in Australia	Range: 6–36
< 15	4 (40)
15~30	3 (30)
≥ 30	3 (30)
Prediabetes diagnosed after immigration	
Yes	9 (90)
No	1 (10)
Duration of prediabetes diagnosis (years)	Range: 1–18
Household composition	
With spouse and children	5 (50)
With spouse only	1 (10)
Alone	1 (10)
With adult children	2 (20)
With spouse, children and grandchildren	1 (10)
Health insurance	
Medicare plus private	9 (90)
Medicare only	1 (10)
Religion	
Christianity	2 (20)
Buddhism	2 (20)
No religion	1 (10)
Not reported	5 (50)
Religious dietary rules	
Yes	1 (10)
No	9 (90)
Oral antidiabetic medication	
For prediabetes	1 (10)
For type 2 diabetes	2 (20)
None	7 (70)

### 5.2 Thematic Findings

**FIGURE 2:**
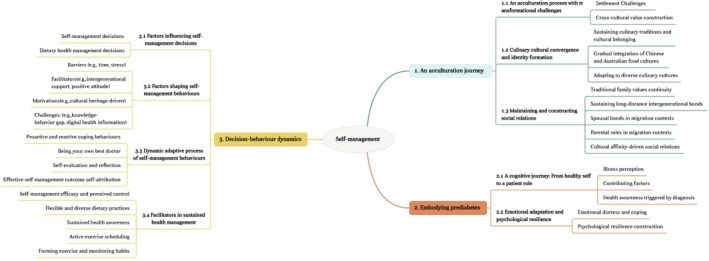
Thematic map illustrating themes and subthemes of prediabetes self‐management experiences among Chinese‐speaking immigrants in Australia.

Three themes relating to immigration and prediabetes self‐management in participants' immigrant country, Australia, emerged from the data: (1) An acculturation journey, (2) Embodying prediabetes and (3) Decision‐behaviour dynamics. These themes were explored and the analysis was further developed, drawing on the conceptual model of the process and outcome of empowerment illustrated in the PEM, addressing individual adaptation, cultural integration and behavioural change processes. The thematic map is illustrated in Figure 2.

### 5.3 Theme 1. An Acculturation Journey: Reshaping Cultural Identity and Social Connections in Immigrant Lives

#### 5.3.1 An Acculturation Process With Transformational Challenges

Participants transformed their lives as they faced and addressed settlement challenges while reconstructing their cultural values. Their acculturation journey progressed from initial environmental exploration and living difficulties to the integration of cross‐cultural values.

##### 5.3.1.1 Settlement Challenges

After relocating to Australia, participants experienced three phases of settlement: initial exploration of a new environment, early to mid‐term living challenges and later stage acculturation hardships. From the initial ‘novelty effects’ to early and mid‐term living, challenges mainly related to practical adjustment issues, such as adapting to a new lifestyle, a decline in social status, regional disparity and reduced purchasing power (‘same money, different worth’).

We still had to adapt to life here… Back in Indonesia, I had 4 maids at home, but here I had to do everything myself… We couldn't afford that here, it's very expensive. [P07]

The later‐stage challenges mainly involved acculturation hardships, including barriers to cross‐cultural interactions, food adaptation challenges, different cultural norms and ongoing language issues.

The food culture and socializing with others. These days, Westerners and Chinese people, most still tend to hang out with their own groups… The challenges are very big. It takes a lot more effort and energy to accept and really [fit] in with their [local] cultural customs. [P05]

##### 5.3.1.2 Cross‐Cultural Value Construction

Participants perceived and adapted to divergent social values as they navigated cross‐cultural transitions. Exposure to Australian values that contrasted with participants' pre‐immigration cultural perspectives, including casual social culture, adolescent autonomy, social aesthetic standards, social equality and religious diversity, shaped their evolving value system. While participants experienced various cultural differences and held varying views on local social equality, they developed an inclusive value system, as illustrated by Participant 5's narrative of religious integration and reflections such as ‘You cannot say mine is good and yours is bad’ and ‘We cannot simply judge which country is better’.

#### 5.3.2 Culinary Cultural Convergence and Identity Formation

Participants navigated cultural identity through culinary practices, creating hybrid dietary approaches that balanced emotional connections to traditional foods while adopting new foods that were easily accessible.

##### 5.3.2.1 Sustaining Culinary Traditions and Cultural Belonging

Nine participants described how their culinary culture contributed to both their sense of traditional cultural belonging and their integration into Australian culture. Participants expressed deep emotional ties to their traditional cuisine, for example, ‘the dumplings tasted very similar to those my mom used to make, I love them so much’ and ‘when returning to [my] hometown, I binge on all those specialities’.

##### 5.3.2.2 Changing Food Choices

Following immigration, participants gradually embraced a daily diet that blended Chinese and Australian cuisines while also adapting to a diverse range of global cuisines.

Here [in Australia], bread and milk are convenient, but back then [the initial immigration period], we wouldn't eat them… We still cooked rice for breakfast. Now it's changed… now breakfast is a slice of bread, a glass of milk… I learned to make sushi, pasta, and pizza. [P05]

##### 5.3.2.3 Religious Culinary Culture

Four participants had religious affiliations, which had minimal influence on dietary practices, except for one Buddhist participant who continued to observe vegetarian practices during religious festivals.

#### 5.3.3 Maintaining and Constructing Social Relations

Participants established new social connections while preserving family bonds. Half of the participants described how family connections and *traditional family values* continued to be influential. These included educational beliefs, fertility views, filial piety, family responsibilities, career perspectives and fatalistic attitudes. For example, one participant's parents' fatalistic attitudes influenced the participant's attitudes to their everyday diet.

I was subconsciously influenced by them [my parents]. Their attitude about eating was basically that you don't have to watch what you eat at all, that a person's fate is predetermined by heaven. [P06]

The relational networks for participants were characterised by multi‐layered support structures from family members, peers and community networks. They sustained *long‐distance intergenerational bonds* with ageing parents in their homeland through emotional and financial interactions. These transnational family connections notably influenced health behaviours, with some participants following their parents' guidance on blood glucose control despite geographical separation.

My mom often tells me things on WeChat, like if you're older, the blood sugar standards can be a bit more relaxed. That's what she says. Anyway, she's kind of like a knowledge asset to me, because she maintains good [blood sugar] control. [P04]

However, these intergenerational bonds also created tensions around health management despite ongoing emotional connections.

My parents really dislike [controlling blood sugar], they don't take it seriously at all. When I talk about it, I get rejected… Whenever I talked about wellness, healthy eating, or dietary precautions, my mother would nearly lose her temper. [P06]

##### 5.3.3.1 Spousal Bonds in Migration Contexts

Immigrants who relocated together relied on each other for primary support, while others faced challenges balancing cross‐cultural marriages or managing relationships with partners still residing in their homeland.

My husband is Muslim…the two of us are so different. He uses very primitive methods, traditional ways, while I want to use the most advanced methods… Something my husband uses, sometimes I secretly drink it now. Why? Because I've realized these traditional methods might have their reasoning. Recently, I discovered he's been secretly eating my health supplements…[Int: You mean both of you have become somewhat integrated with each other?] Yes, yes, yes, I think it's quite good. [P02]

##### 5.3.3.2 Parental Roles in Migration Contexts

Parental roles in migration contexts illustrate how parenting and intergenerational relationships are transformed in transnational settings. Participants navigated significant changes in their parenting roles as they balance traditional Chinese values with Western norms.

My eldest daughter came here after graduating from elementary school to study here. In school, I was really worried her English might not be good… As parents, we really need to be able to accept their [Australian] cultural customs, and it takes a lot of effort and energy. [P05]

##### 5.3.3.3 Cultural Affinity‐Driven Social Relations

Most participants demonstrated strong engagement with Chinese communities. Their cultural affinity was evident in both their residential preferences (within the Chinese‐speaking community) and social participation, such as engaging in cultural dance groups, volunteering at community language schools and supporting culturally affiliated council candidates. These cultural gatherings facilitated discussions and the sharing of health‐related information. For example, one participant narrated that several members of her dance group had diabetes, and they often shared thoughts about dietary management during their coffee breaks after dancing [P05].

### 5.4 Theme 2. Embodying Prediabetes: Cognitive Reconstruction and Emotional Adaptation in the Transition to Patient Role

#### 5.4.1 A Cognitive Journey: From Healthy Self to a Patient Role

Participants underwent a process of cognitive reconstruction, primarily experiencing a transformation from perceiving themselves as a healthy person to becoming a patient with prediabetes. They identified the factors related to the onset of their prediabetes as genetic, metabolic and unhealthy lifestyle factors, but they also believed that psychological stress and a lack of health knowledge were significant contributing factors.

I feel that I was diagnosed with prediabetes when I was under the most stress… Those three years when I was running my business were my toughest time, that's when I developed it. [P07]

Basically, I just go from the office to the car, from the car to home, it's just two fixed points. Or when I attend activities, it's always going out at night to eat, eat, eat, eat, eat. And then, you know, staying up late ‐ isn't it just these things, right? And then there's something even more important, which is actually having absolutely no concept about health or proper diet. [P02]

This recognition of contributing factors often emerged alongside a significant shift in *health awareness triggered by diagnosis*. Half of the participants reported that their diagnosis catalysed a shift in life priorities, such as shifting from the ‘two fixed points’ lifestyle to a ‘health comes first’ perspective.

Now I feel that health comes first…Even if you have everything else, if you don't have health, what use is it? It's completely useless, right? [P02]

#### 5.4.2 Emotional Adaptation and Psychological Resilience

When faced with a diagnosis of prediabetes, participants experienced a series of cognitive, emotional and psychological transformations, developed balanced health perspectives, and gradually constructed psychological resilience. All participants described a three‐stage psychological transition following a diagnosis of prediabetes. Their initial response at diagnosis was anxiety, shock and distress, with four participants feeling at a loss and two conducting ‘compulsive’ daily blood glucose self‐monitoring at mealtimes. Post‐diagnosis adjustment varied from denial to acceptance of the diagnosis, followed by adopting a positive mindset and initiating self‐regulatory behaviours. However, many had ongoing concerns, fearing disease progression and hypoglycaemia, and feeling like they were ‘treading on thin ice’. Some also experienced intense health anxiety

This [worry] is still lingering there, vaguely…I can't imagine what could make me suddenly [pass away] … How high would my blood sugar have to be, or what blood sugar level could make me go to sleep tonight and never wake up… [P04]

Participants coped with these emotional responses through knowledge acquisition, medical consultations, attention diversion and stress relief strategies such as music and shopping. Moreover, *psychological resilience* was constructed through self‐reconciliation, self‐reflection, mindfulness, traditional beliefs and religious faith, as participants viewed themselves as ‘The Unkillable Cockroach’ and adopted the mindset of ‘turning a misfortune into a blessing’.

You need to keep a more positive attitude, don't always think of yourself as a sick person, then say, “I must care about this, care about that…” You don't need to be so worried. [P05]

I feel that having this thing [prediabetes] has helped me learn about health [laughs], a blessing in disguise. Without this thing, I wouldn't have paid any attention to my health at all. [P01]

### 5.5 Theme 3. Decision‐Behaviour Dynamics: Mapping Agency and Adaptiveness in Self‐Management Processes

#### 5.5.1 Factors Influencing Self‐Management Decisions

*Self‐management decisions* involved participants weighing personal values, social influences and practical constraints, as illustrated in Table 1. Most participants who had co‐morbid conditions underestimated the seriousness of prediabetes and prioritised their other health conditions. Recommendations from healthcare professionals influenced their decision‐making, particularly during the initial post‐diagnosis period. Various psychological factors, including external attribution tendency, perceptions of risk decline, risk‐averse mindsets and personal determination, collectively shaped their self‐management decision‐making.

*Dietary health management decisions* were separated as a standalone category, reflecting their emergence from the complex integration of family preferences, nutritional knowledge and practical considerations within the household. Individual and family preferences played a fundamental role in dietary choices, shaped by Chinese meal traditions, regional food habits, personal preferences and the preferences of their children and partners. The family cooking system served as a crucial determinant, where the primary cook, cooking skills and established culinary styles significantly shaped their daily diet. Moreover, dietary decision‐making was influenced by their acceptance of dietary changes, subjective judgement of food's nutritional value and considerations of the glycaemic index (GI) (Table 2).

**TABLE 2:** Factors influencing self‐management decisions.

Code	Examples of participant quotes
(1) Self‐management decisions	
Code 1.1 Construction of personal health priorities	‘These two problems [high cholesterol and iron deficiency] are more prominent than the blood sugar issue, so the blood sugar issue wasn't even mentioned in my daily schedule’. [P06] ‘Stomach, prostate, eh, that's right. I'm mainly more concerned about these two. [Interviewer: You mean these two issues are in your priorities?] Yes, yes, yes, with blood sugar, I mainly just pay a bit of attention to it day‐to‐day. I feel like it's probably not a big problem’. [P08]
Code 1.2 Health observations in social networks	‘Back when I was studying at UWA, there was this local girl who was very overweight and had diabetes. She was constantly giving herself injections every day…In my family, I've got two cancer patients… they're really close relatives, you know, so for me, health comes first now’. [P02] ‘Lately I've been participating in more activities and seeing many people with problems caused by diabetes. It's such a pity, it's so serious, so serious. You feel really shocked—how did they end up like this?…He carries a needle with him all the time, and whenever he feels unwell, he immediately injects himself. I think to myself, how could I let myself get to that state?’ [P05]
Code 1.3 Peer advice	‘I listened to that friend. It was his suggestion. I just took his word for it and went and did (the glucose tolerance test) …’ [P04]
Code 1.4 Professional recommendations	‘So after the doctor checked me out, I just followed what he told us to do, right, so that's when I also started to control things’. [P05] ‘Later, after what the doctor said, around 2021, roughly, after that, I just paid a little more attention, you know, but didn't really restrict my food’. [P09]
Code 1.5 Psychological factors	‘I felt that after checking again and again, I didn't feel like there were any particularly shocking numbers coming out. So I reduced the frequency… How can I maintain these improvements from the first half of the year? But I'm also thinking, well, maybe I can deal with it. Anyway, I am definitely going to figure out a solution’. [P04] ‘I thought to myself, ‘Would this (Doctor‐recommended Ozempic) be bad for my body?’ Right? I was thinking about the side effects, so I never went to get this injection… This (eye check) was normal, so I went once, then a second time, and after that, I didn't go anymore because I felt it was normal anyway’. [P10]
Code 1.6 Autonomous cognition and preferences	‘If you tell me to run 5 km every day, or to do some kind of exercise here, I just won't do it. But let's start with twice a week—that's doable…I can do it (exercise) myself, it's not that I must be accompanied by my husband or friends… if someone keeps pushing me to go, I won't go’. [P10]
Code 1.7 Convenience and accessibility of strategies and resources	‘There's a key thing that always makes me want to take shortcuts…It's not like I deliberately tried, it was probably just to save effort. I can only accept being told what not to eat; then I won't eat it and will eat something else instead. Fine, what else can I eat? Just tell me… Now I put everything in the oven. I found it's quite convenient. Now I don't use oil at all’. [P04]
Code 1.8 Financial considerations	‘He (GP) asked if I wanted to get my feet checked… I said no… he said this foot examination was free, ‘I'll write you a referral,’ then I went ahead and did it’. [P10]
(2) Dietary health management decisions	
Code 2.1 Individual and family preferences	‘Exactly! Fuzhou snacks, Fuzhou snacks. Those are still our old habits; we still really love eating those [laugh]’. [P08] So now for Chinese meals, I always eat sweet things, like sometimes pastries or desserts with meals. [P07] ‘My husband and I both eat quite healthy now, um, as for the children, they still like to eat meat… I had a period when I ate too much beef with them in the evenings… The doctor said, your cholesterol is a bit high…’ [P02]
Code 2.2 Food selection based on glycaemic index (GI)	‘Basically, I just look at things simply. Usually, if I've seen something has a high GI, I eat less of it. And then whatever has high carbs, Things like sugar, honey, and fruit all belong to the same category—they all contain relatively high sugar content, so I control them myself, just like that’. [P03] ‘I'm very careful with fruit. I check whether the so‐called GI is high or not. They say watermelon is very high (in sugar), so I eat a little less of it. Pomegranate is high too, so we eat a bit less of that…I've tested it (the GI of sweet potatoes)…Well, the cooking method affects it, so I mostly eat them raw’. [P01]
Code 2.3 Family cooking system	‘I always tell him (my husband) that cooking so little isn't enough, you need to cook more. He actually cooks a big pot already, a big pot full of potatoes, you know? And the meat, he doesn't put in that much. He puts it in bags to cook, but if it were me, I'd prefer less potato and more meat [laughs loudly]’. [P02] ‘For meals, generally, whatever she (my wife) cooks, I eat whatever she makes’. [P08]
Code 2.4 Perceived nutritional value of foods	‘My staple food is still bread. I need its fibre, that is, the dietary fibre that's in it. When I look at the nutritional proportions, I think bread can replace many of the grains that should be in rice. Of course, I also check rice's nutritional content. If something is lacking, I might supplement it a bit’. [P01] ‘Sometimes I occasionally drink some honey water. It's supposed to be better for the throat’. [P06]
Code 2.5 Acceptance toward dietary changes	‘Like with rice, switching to whole grain toast, that I can handle, but vegetables, I won't stir‐fry vegetables to eat, just like that… Vegetables are where I have difficulty. Fruit is still okay, we eat fruit anyway, but vegetables I find (difficult)’. [P10] ‘I don't really like bread much. But now I'm eating this bread. The main foods are pretty boring—just sticking to this bread. And sticking to that Indian rice. I've started eating things I didn't really like before, stuff I rarely ate’. [P04]

#### 5.5.2 Factors Shaping Self‐Management Behaviours

Self‐management behaviours were shaped by factors detailed in Table 3. These factors operated at multiple levels, from structural barriers and facilitators to individual motivations and challenges, often interacting in complex ways to shape the participants' experiences in managing prediabetes. *Barriers* to self‐management occurred at different levels across individual, daily life, cultural and health access‐related factors. Key barriers included, but were not limited to, healthcare‐seeking choices (e.g., limited access to specialists), daily management challenges (such as cooking and food access), medication adherence and personal factors.

**TABLE 3:** Factors shaping self‐management behaviours.

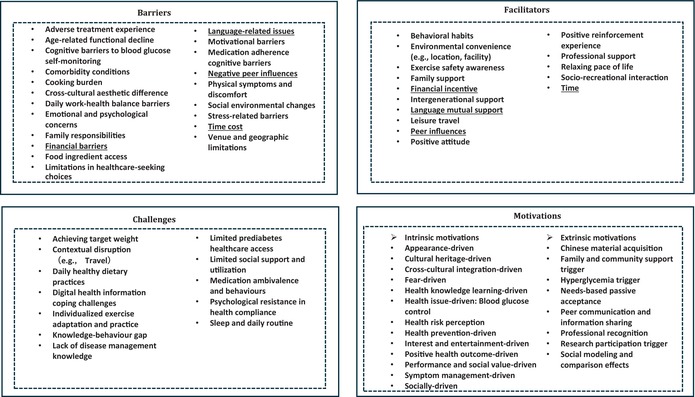

*Facilitators* represented supportive conditions that made self‐management more accessible and achievable. They were predominantly characterised by strong social support networks and lifestyle factors that promoted well‐being, such as leisure travel and a relaxing pace of life. Notably, factors such as peer influences, language, time and financial resources demonstrated dual effects. Five participants identified peer influences as facilitators of exercise and weight management, while three participants reported negative peer influences; for example, excessive focus or exaggerated information can increase disease‐related anxiety.

I usually listen to his [friend] advice during the day, but at night I don't dare to—he often says things that scare me so much I can't sleep, you know?… [feel] very anxious. [P04]

Language also had dual effects: onsite Chinese interpretation and peer language support facilitated prediabetes self‐management, while communication barriers in healthcare settings and inadequate translation services hindered it. Medical English barriers were prominent among all participants regardless of their English proficiency levels.

When it comes to disease management and control or certain medical terms, even people with a good command of English may not understand. [P01]

*Motivations* reflected both intrinsic health values and extrinsic social and contextual factors that drove participants' engagement in self‐management. Intrinsic motivations were primarily health‐oriented, including acquiring health knowledge, outcome‐driven, prevention‐driven and risk perception, while at the same time including cultural integration, cultural heritage and social aspects.

This activity [prediabetes self‐management] lets us reach out, right? We can socialize; we can reach out. [P10]

Extrinsic motivations were triggered by various factors such as professional recognition, peer communication, social modelling and comparison and social support. Social modelling and comparison illustrated how individuals motivated their self‐management behaviours through both modelling behaviours (following others' examples) and contrasting references (using others as reference points for self‐comparison).

He [my friend] is very careful now, very mindful about everything. He controls his portions even better than I do—very vigilant. He absolutely never overeats, while sometimes I still can't manage that. [P01]

*Challenges* in self‐management were identified across multiple domains, ranging from the individual level to the practical and organisational levels. Almost all participants reported limited access to social support (e.g., family support, social activities and immigrant health information resources) and faced daily dietary challenges such as resisting food temptations.

If there's some delicious‐smelling food just sitting right there, I'm gonna dig in—no holding back [laughs]. [P08]

Many participants demonstrated a knowledge‐behaviour gap in their prediabetes self‐management, where they understood what they needed to do but struggled to consistently implement them.

I knew all these things, but sometimes I just couldn't control myself… but I often forget about it. Actually, I think everyone understands these principles. [P06]

Participants described challenges in navigating digital health information due to the overwhelming volume and difficulty in evaluating online resources. Older participants reported additional barriers, such as limited digital literacy, poor eyesight and vulnerability to misinformation on social media.

My eyes aren't good anymore. When I go online, all those tiny words going “ding ding ding” make me dizzy. [P09]

#### 5.5.3 Dynamic Adaptive Process of Self‐Management Behaviours

This subtheme revealed how individuals exercised agency and adaptiveness in navigating their health management through reflective practices, adaptive coping strategies and self‐efficacy attributions.

##### 5.5.3.1 Proactive and Reactive Coping Behaviours

Participants utilised both anticipatory strategies to prevent fluctuations in blood glucose levels and made responsive adjustments when issues arose. All participants talked about their proactive and reactive coping behaviours. These behaviours primarily included health information seeking (*n* = 10), dietary adjustments (*n* = 10), glucose self‐monitoring (*n* = 9), exercise strategies (*n* = 10) and multi‐channel health intervention seeking (*n* = 7). Most participants reported engaging in proactive digital health information seeking, highlighting their view that the ‘Internet is an excellent doctor’. Some participated in community lectures, engaged in independent study of dietitian courses and sought guidance from pharmacists to enhance their knowledge and skills.

Four distinct dietary adaptation patterns were identified. The first pattern involved avoidance strategies, like not buying sweet foods and avoiding the preparation of staple foods. The second pattern showed proactive coping strategies that included managing hunger, controlling portions, eliminating soft drinks and diversifying food options. The third pattern demonstrated balanced adjustments; for instance, choosing to replace rice porridge with mung bean porridge, striking a balance between ingredient swaps and taste preferences. The fourth focused on modifying cooking behaviours, particularly shifting from frying to baking, minimising oil usage and adjusting traditional recipes to align with health guidelines.

Most participants opted for low‐intensity activities, primarily walking, with frequency ranging from 2 to 7 times weekly, but only two did this regularly while others generally adopted irregular patterns or substituted structured exercise with activities of daily living. Multi‐channel health intervention‐seeking behaviours included dietary supplementation, complementary and alternative therapies and cross‐border healthcare. Weight management included goal‐setting for weight loss with their GPs (*n* = 3) and dietary management such as intermittent fasting, meal replacement and low‐fat/low‐carb diets (*n* = 3). Blood glucose monitoring patterns varied: self‐initiated regular monitoring (*n* = 3), self‐initiated irregular monitoring (*n* = 4), professional monitoring by healthcare providers (*n* = 2) and no monitoring (*n* = 2).

##### 5.5.3.2 Being Your Own Best Doctor

Participants developed a level of confidence and actively engaged in self‐directed health management. They often viewed themselves as their own best doctor, which was reflected in their proactive engagement (*n* = 8) as seeking individualised blood glucose standards, proactively proposing personalised medication adjustments to their GPs, and establishing self‐defined health criteria. They selectively adopted health information provided by online sources or healthcare professionals.

Sometimes when there are explanations about prevention, I'll take a look. But I don't necessarily follow everything in the letter. If I find something suitable, I'll take it on board, otherwise… [stops talking, laughs]. [P07]

Ongoing *self‐evaluation and reflection* were evident through participants' personal health literacy and their self‐assessment of their levels of physical activity, sleep patterns, dietary patterns, medication effects and blood glucose control. Notably, participants identified significant gaps in their physical activity engagement: complete inactivity (*n* = 2), irregular participation (*n* = 2), insufficient intensity or volume (*n* = 2), sedentary behaviour (*n* = 1) and lack of intentional activity (*n* = 1). With respect to their diet, seven participants recognised and articulated their healthy eating habits such as a good vegetable intake, maintaining food diversity and controlled portions; and they also identified the influence of unhealthy dietary habits such as recognising how nighttime eating potentially influences morning fasting blood glucose levels. In the self‐perceived medication effects, among the three participants who had taken metformin for their blood glucose control, two reported positive outcomes, while the other considered their current blood glucose control was unrelated to the medication.

*Effective self‐management outcome self‐attribution* indicated participants' recognition and understanding of improvements in their health. While most participants attributed their success in controlling weight and blood glucose to lifestyle changes and personal persistence, others believed that the reason for their weight loss was the use of supplements and metformin.

#### 5.5.4 Facilitators in Sustained Health Management

Facilitators in sustained health management identified factors that helped individuals to maintain self‐management behaviours over time, including psychological control, behavioural flexibility, sustained awareness and structured routines. *Self‐management efficacy and perceived control* were demonstrated through their confidence in sustaining lifestyle changes and a sense of self‐satisfaction. Participants described adopting *flexible and diverse dietary practices* by balancing food texture, dietary diversity and value for money. In addition, a relaxed self‐management approach, demonstrated by a balanced rather than restrictive attitude (‘occasionally having some is fine, just try to eat less’ [P08]), coupled with the convenience brought by dietary changes (‘I switched to eating bread. I find it quite convenient as I don't need to cook’ [P10]), promoted adherence. In terms of healthcare management, participants emphasised *sustained health awareness* and established systematic health maintenance habits through *active exercise scheduling* and *forming exercise and monitoring habits*.

I know I'm on the borderline, so I pay attention to food—eat less sweets and rice and more vegetables and fruits. [P08]

## 6 Discussion

Through immersion in reflective documents and data, this study found that managing prediabetes among Chinese‐speaking immigrants in Australia was characterised by a strong sense of ‘self‐reliance’, which was inextricably linked to their migration experiences. We identified how underlying cultural values (Section 5.3: Theme 1) and the experience of a prediabetes diagnosis (Section 5.4: Theme 2) jointly shaped participants' self‐management strategies (Section 5.5: Theme 3). These findings illustrate how participants' empowerment outcomes (identity formation, decision‐making and self‐management behaviours) emerged from the dynamic interplay between internal processes (cultural adaptation, health perceptions, emotional responses, information‐seeking and coping strategies) and external social network reconstruction based on the PEM (Small et al. 2013).

### 6.1 Culture and Beliefs

First‐generation immigrants typically retain their native language and cultural values from their country of origin (Zhang et al. 2024). This was evident among study participants throughout their settlement journey. While initial struggles centred on basic living necessities, later stages involved deeper issues of cultural adaptation and social integration. Even after decades of settlement in Australia, participants had managed to preserve their original cultural practices and dietary traditions while navigating the process of cultural integration. This cultural preservation was reflected across values, language, diet, social interactions and intergenerational relationships, which in turn impacted prediabetes self‐management. This aligns with research on Javanese with type 2 diabetes, where traditional beliefs were found to significantly influence the perceptions and response to health information and self‐management behaviours (Sari et al. 2022).

Participants' cultural adaptation reflected a unique journey of empowerment, where they maintained their cultural identity while continuously adapting and learning, ranging from initial recognition of cultural differences to gradually finding a way to blend and balance different cultural experiences. During the internal cognitive adaptation, participants encountered cultural differences between Chinese and Australian perspectives, primarily across value systems, healthcare philosophies and dietary cultures. Through cultural navigation, participants gained cross‐cultural insights and achieved balance by constructing an inclusive value system, utilising words such as ‘casual’, ‘inclusive’, ‘neutral’, ‘independent’ and ‘becoming calm in the face of illness’. Such cultural integration enabled some participants to develop integrative strategies that harmonised traditional health beliefs with Western healthcare practices.

Unlike other populations where religion can act as a facilitator for diabetes self‐management (Zhang et al. 2024), religious involvement had limited influence on self‐management behaviours among this study population. Rather, Chinese Philosophy (e.g., Confucianism, Taoism, Legalism and Mohism) appeared to have a broader influence on health beliefs and behaviours. Traditional beliefs, particularly those emphasising achieving harmony and maintaining balance such as ‘tianren heyi’(unity of heaven and humanity) and ‘yin–yang balance’, may, to some degree, explain participants' integrative health approaches, including blending Traditional Chinese Medicine (TCM) and Western perspectives, and adopting culturally blended dietary approaches (Chen 2001; Meng et al. 2024; Osokpo and Riegel 2021). This reflects the subtle, often unconscious influence of Chinese philosophy on health‐related behaviours, regardless of religious affiliation or geographical location, underscoring the importance of recognising cultural heritage in care delivery (Chen 2001; Meng et al. 2024).

### 6.2 Language

Similar to other CALD groups, language is a persistent barrier for first‐generation Chinese immigrants. In daily life, language issues significantly constrained their health‐related choices when shopping for food, dining out, accessing English‐language websites and interacting cross culturally. Medical language barriers, unlike general immigration‐related language challenges, were evident across all levels of English proficiency. All participants preferred native language materials for managing their prediabetes. This reflects a commitment to cultural identity that extends beyond mere communication challenges.

Language played a dual role in prediabetes self‐management: while English‐proficient Chinese peer support and onsite interpretation services facilitated self‐management, communication barriers in healthcare settings and inadequate translation services posed considerable challenges, particularly regarding quality and accessibility, which is consistent with previous research (Khatri and Assefa 2022). While helpful, these services were often culturally inappropriate and required extra time to arrange interpreters (Khatri and Assefa 2022). Information about these services failed to reach Chinese immigrants because they were not promoted through preferred channels such as Chinese community centres or popular social media. Building on this, expanding translation services to include peer and family support presents a viable solution (Khatri and Assefa 2022). Gaps in bilingual health information resources remain a significant challenge. Healthcare providers need to adopt plain language and ensure immigrant persons with prediabetes have access to culturally appropriate materials (Zhang et al. 2024).

### 6.3 Diet

Internal (nutritional judgements, individual preferences, acceptance of change) and external (family preferences, cooking systems, GI of foods) empowerment processes collectively shaped participants' dietary decisions for managing their prediabetes, ranging from passive avoidance to balanced adaptation and proactive coping strategies. These individual‐level strategies were typically insufficient without support, which has been shown to be an important aspect of making sustained lifestyle changes (Abel et al. 2018, 2021). Access to traditional foods and adapting to new foods was a challenging aspect of participants' cultural adaptation after immigrating, and this became more challenging when prediabetes was diagnosed. They experienced cognitive dissonance between increasing health awareness and emotional attachments to traditional foods. For example, when advised by their GP to ‘quit rice entirely’, which might seem to be a minor dietary change, some participants expressed strong emotional attachments to rice, describing it as an ‘addiction’. This mirrors findings that Chinese Americans with type 2 diabetes found it ‘difficult to remove such a critical cultural staple from their diet’ (Li‐Geng et al. 2020). However, for some foods like rice, different cultural eating practices and individual acceptance need to be considered before imposing universal dietary restrictions, given the cultural importance and high‐quality evidence shows only a weak association between white rice consumption and diabetes risk in Chinese populations (Bhavadharini et al. 2020).

### 6.4 Emotional Distress

Emotional adaptation and psychological resilience reflected ‘accepting diagnosis’ and ‘acknowledging unchangeable reality’ in internal empowerment, signalling their transition from being a passive recipient to active self‐management. The resettlement process in Australia significantly impacts the mental health and well‐being of immigrants (Staneva and Chai 2025), and study participants experienced similar challenges; some considered immigration‐related stress, stemming from social status decline, regional disparity and difficulties in settlement, to be significant factors that contributed to the development of their prediabetes. After diagnosis, participants faced increased psychological stress, particularly in relation to making and adhering to dietary and physical activity changes and medical follow‐ups. They viewed ‘dietary modification’ as a forced change to existing habits, involving the consumption of disliked or unfamiliar foods, which often triggered resistance and negativity. This finding aligns with Lee et al. (2022), who found that Chinese immigrants to Australia, having migrated less than 10 years prior and without prediabetes, resisted dietary changes, especially regarding unfamiliar foods (Lee et al. 2022).

People with prediabetes commonly experience fatigue, anxiety and depression (Wicke et al. 2024), with approximately 11% more likely to experience depression compared to individuals with normal blood sugar levels (Chen et al. 2016). Consistent with these findings, our participants reported psychological challenges such as diabetes fear, anxiety and a sense of loss from lifestyle change. These complexities were further heightened by navigating cultural transitions as immigrants. Unfortunately, they reported experiencing a lack of psychological support and feeling their emotional needs were neglected by healthcare providers. This neglect is particularly concerning given that the combination of prediabetes and depression/anxiety symptoms shapes the risk of diabetes (Bell et al. 2020; Wicke et al. 2024).

Notably, providers' communication styles significantly impacted participants' psychological comfort. This was exemplified by several contrasting healthcare experiences: positive interactions (e.g., providers' use of humour) reduced anxiety, while negative encounters, marked by alarming responses, exacerbated feelings of distress. When external support was limited, participants gradually built psychological resilience through internal empowerment, creating a foundation for active prediabetes self‐management. However, ongoing psychological and emotional support remains vital, particularly given the complex emotional phases that individuals may encounter, ranging from the challenges of immigration to the adjustments required following a diagnosis (Bell et al. 2020; Staneva and Chai 2025). Therefore, accessible channels for psychological support and stress management guidance for Chinese immigrants living with prediabetes need to be established, aligning with the Australian joint position statement (Bell et al. 2020).

### 6.5 Relational Support

Relational support demonstrated reconstructed social networks in post‐immigration settings as a vital external empowerment process. Family support was the primary layer, reflecting unique patterns spanning both local and distant relationships. This aligns with Hook and Glick's (2020) observation that migration reshapes family structures (Hook and Glick 2020). Unique challenges arose in cross‐cultural families, where different health practices required integration and families were geographically separated. Second‐generation immigrant children who are well‐adjusted to the host culture often served as cultural mediators. This means that they respect their parents' autonomy while facilitating acculturation experiences through procedural guidance, medical navigation, communication bridging and interpreting cultural norms (Subramoney et al. 2025). In this study, most participants (*n* = 7) had second‐generation immigrant children, of whom adult children actively helped them navigate the healthcare system. Additionally, long‐distance bonds with parents in participants' homeland influenced their health perceptions and practices through parents' health beliefs and shared experiences, consistent with existing literature on transnational family support (Zhang et al. 2024).

Peer support emerged as another crucial layer, with local and long‐distance peer connections shaping health cognition and behaviours. Peer influences have mixed effects on health behaviours: peers' health achievements, such as running accomplishments, helped maintain exercise routines, while excessive or exaggerated health information from peers triggered anxiety and emotional contagion. This highlights the need for health literacy and skills to evaluate peer‐shared information. Health professionals should take a more active role in education, helping individuals filter information, enhance health literacy and avoid content that may cause unnecessary panic (Lin et al. 2024). Furthermore, community networks emerged as a rich third layer of support, where cultural affinity naturally shaped social connections and engagement patterns. This cultural connection served as the foundation for social networks among Chinese immigrants, naturally drawing participants toward those who shared a similar cultural understanding. Culturally sensitive activities and venues enhanced community engagement by integrating traditional elements (e.g., traditional dietary wellness workshops, traditional dance classes), aligning with Theodosopoulos et al.'s (2024) findings on the importance of cultural concordance in immigrant healthcare delivery (Theodosopoulos et al. 2024).

### 6.6 Behavioural Dynamics

A significant knowledge‐behaviour gap emerged across self‐management behaviours, particularly with physical activity behaviours. Participants often struggled to translate this knowledge into sustained action, despite understanding lifestyle recommendations and growing awareness of the need for behavioural change. Various external constraints and personal confidence levels influenced this gap. Individual intentions to change behaviours are notably affected by real‐world factors, such as work commitments and perceived self‐efficacy. Work and home responsibilities significantly hinder the implementation of self‐care, while confidence in health decision‐making helps turn intentions into sustainable lifestyle changes (Jokar et al. 2025).

Many participants attributed their success in self‐management to persistence at making dietary and physical activity changes, reflecting enhanced personal control and agency, whereas only one individual with prediabetes primarily attributed improvements to medication. This was consistent with participants' overall attitudes toward treatment. Most participants viewed medication as unnecessary and preferred lifestyle interventions unless they progressed to diabetes, as has been observed in other studies (Ren et al. 2023). Moreover, participants reported behavioural changes that supported their long‐term management of prediabetes. Several key facilitating factors emerged that had contributed to their achievement of sustained health management. Regular physical activity was facilitated and supported by various interacting factors: initial concerns about blood sugar triggered action, while convenient facilities, established routines, a positive mindset, habit formation and visible health improvements reinforced the ongoing commitment. This progression illustrated participants' transition from reactive to proactive health management, where physical activity evolved from a medical recommendation to an integrated aspect of daily living.

Almost all participants actively sought digital health information for their prediabetes self‐management, viewing the internet as a valuable ‘alternative doctor’. Nonetheless, participants faced several challenges in acquiring digital health information, mirroring the digital literacy barriers faced by immigrants identified in previous reviews (Estrela et al. 2023). These included accessibility issues for older immigrants, a lack of skills for critically evaluating online health resources, confusion and fatigue resulting from information overload and frustration arising from conflicting information sources. Such challenges sometimes resulted in psychological resistance and, in severe instances, negative coping strategies, notably ‘self‐abandonment’.

### 6.7 Limitations

The study was limited to a single geographic location with participants, and all participants were over 40 years old despite broader inclusion criteria. Given that prediabetes is becoming more common among younger individuals, our findings may not reflect the diverse experiences among Chinese‐speaking immigrants with prediabetes. However, the transferability of findings was enhanced through detailed participant descriptions and illustrative quotes, along with thematic maps and theoretical framework comparisons (Stalmeijer et al. 2024). As this study employs RTA based on one‐on‐one interviews, the findings may be influenced by the researcher's interpretative perspective and the inherent limitations of self‐reported data. We addressed potential research biases through rigorous reflexive practices, including systematic documentation, peer debriefing and consumer representative engagement. Nevertheless, this study provides valuable insights into how immigration experiences and cultural influences affect prediabetes self‐management among Chinese‐speaking Australians, offering a foundation for culturally appropriate strategies for prediabetes management and type 2 diabetes prevention.

### 6.8 Theoretical Implications

**FIGURE 3:**
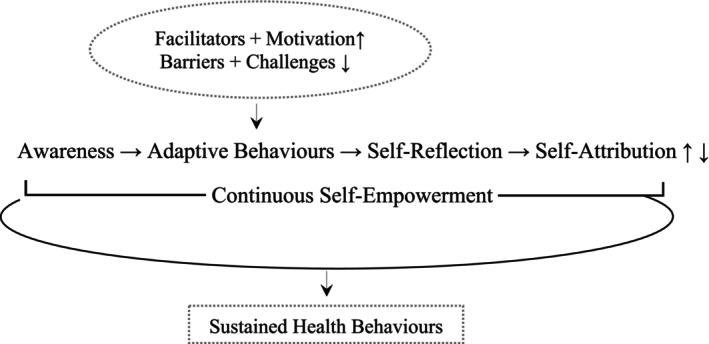
Dynamic self‐management cycle in prediabetes.

This study extends the current understanding of health behaviour changes by identifying a dynamic adaptive process in prediabetes self‐management in Chinese immigrants, illustrated in Figure 3. This process enriches patient empowerment through the interplay of health awareness, strategic behaviours, critical reflection and personal meaning‐making. Adaptive behaviours are shaped by facilitators, barriers, motivation and challenges, leading to sustained health behaviours. Individuals actively reconstruct their health management approach, transforming challenges into opportunities for personal growth and improved well‐being.

### 6.9 Practical Implications

The study highlights a significant gap in awareness of prediabetes among individuals and healthcare professionals (Diabetes Australia 2023). Future strategies should focus on integrating prediabetes into community health promotion and developing culturally targeted intervention programmes. These programmes should adopt a community empowerment approach through evidence‐based content, flexible delivery formats and existing cultural networks, such as potential health education pathways. Individual‐level interventions should incorporate careful consideration of sociodemographic and cultural factors and self‐management influences to ensure tailored care, aligning with previous findings (Zhang et al. 2024).

## 7 Conclusion

The findings from this study describe how Chinese‐speaking immigrants navigate prediabetes self‐management through a dynamic process of cognitive, cultural and psychological reconstruction. Even after decades of settlement, participants experienced persistent language barriers and ongoing acculturative challenges. They developed proactive and adaptive self‐management strategies through a dynamic empowerment process of decision‐making, awareness, adaptive behaviours, self‐reflection and self‐attribution. Adaptive self‐management behaviours were primarily shaped by factors including illness perceptions, emotional distress, cultural values and acculturation, dietary preferences, services and resource availability and local and distant social networks. These findings support the need for future prediabetes management and type 2 diabetes prevention initiatives to focus on empowering both individuals and communities by strengthening self‐management skills, utilising cultural networks and digital platforms while establishing evidence‐based, culturally appropriate materials and communication styles that consider various behavioural influences.

## Author Contributions

Made substantial contributions to conception and design or acquisition of data or analysis and interpretation of data: M.Z., K.C., J.L., L.W. Involved in drafting the manuscript or revising it critically for important intellectual content: M.Z. Given final approval of the version to be published, Each author should have participated sufficiently in the work to take public responsibility for appropriate portions of the content: M.Z., K.C., J.L., L.W. Agreed to be accountable for all aspects of the work in ensuring that questions related to the accuracy or integrity of any part of the work are appropriately investigated and resolved: M.Z., K.C., J.L., L.W.

## Conflicts of Interest

The authors declare no conflicts of interest.

## Supporting information


**Appendix S1:** jan70214‐sup‐0001‐AppendixS1.pdf.

**Appendix S2:** jan70214‐sup‐0002‐AppendixS2.docx.

**Appendix S3:** jan70214‐sup‐0003‐AppendixS3.docx.

## Data Availability

The study data are presented in the article and Supporting Information, with the bilingual version available from the corresponding author upon request.
