# Supplementary figures and images for: Navigating Prediabetes in a Foreign Country: A Qualitative Study of Self‐Management Experiences Among Chinese‐Speaking Immigrants in Australia
Source: J Adv Nurs. 2025 Sep 22;82(6):6332–48. doi: 10.1111/jan.70214 (PMC13176700; doi:10.1111/jan.70214)

# **Supplementary File**
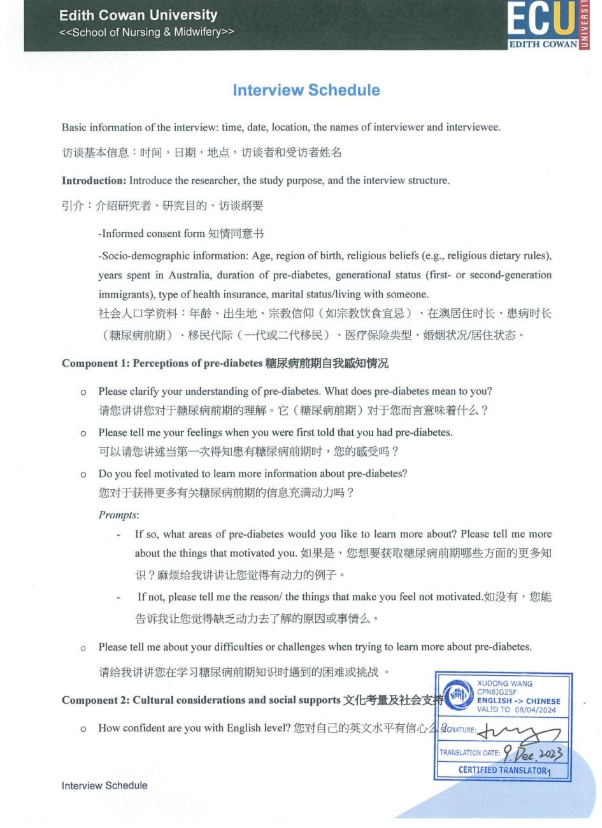


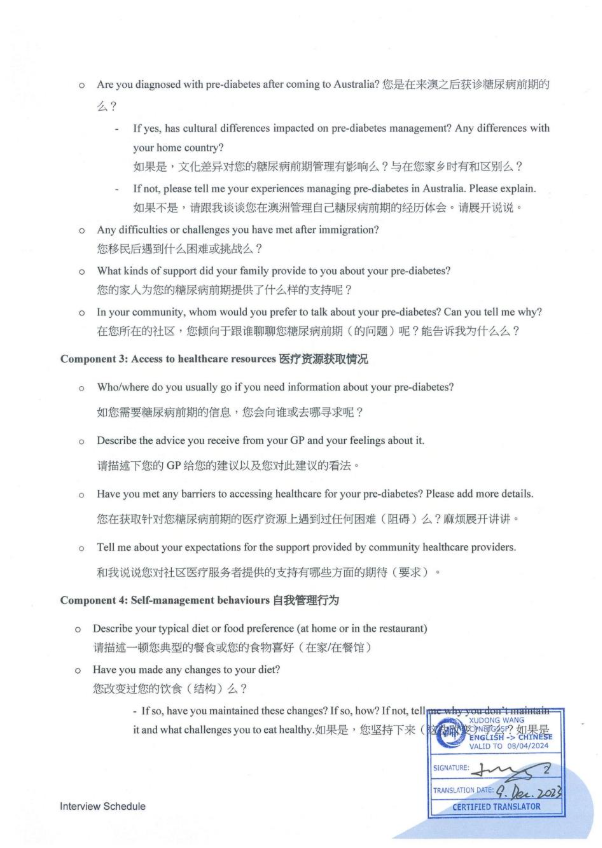

Supplement: Supplementary file 2 — Appendix S2: jan70214‐sup‐0002‐AppendixS2.docx. [file JAN-82-6332-s003.docx]
