# Supplementary material for: Navigating Prediabetes in a Foreign Country: A Qualitative Study of Self‐Management Experiences Among Chinese‐Speaking Immigrants in Australia
Source: J Adv Nurs. 2025 Sep 22;82(6):6332–48. doi: 10.1111/jan.70214 (PMC13176700; doi:10.1111/jan.70214)
Supplement: Supplementary file 3 — Appendix S3: jan70214‐sup‐0003‐AppendixS3.docx. [file JAN-82-6332-s002.docx]

**Appendix III**

**Codes**

**Initial themes**

**Subthemes**

**Themes**

**Patient Empowerment Model**

**Inductive approach**

**Thematic Interpretation**

**Deductive approach**

**Appendix III. Inductive and deductive analysis pathways**
